# 3-(3,4-Dimethoxy­phenyl)-1-(3-pyridyl)prop-2-en-1-one monohydrate

**DOI:** 10.1107/S1600536809013944

**Published:** 2009-04-18

**Authors:** A. Jayarama, Seik Weng Ng

**Affiliations:** a Department of Physics, Mangalore Institute of Technology and Engineering, Badagamijar, Moodabidri 574 225, India; b Department of Chemistry, University of Malaya, 50603 Kuala Lumpur, Malaysia

## Abstract

The pyridyl and aryl rings in the title compound, C_16_H_15_NO_3_·H_2_O, which are located at the ends of the propenone unit, are nearly coplanar with this unit [dihedral angles = 3.74 (14) and 5.06 (13)°, respectively]; the rings are inclined at an angle of 6.2 (1)° with respect to each other. The solvent water mol­ecule forms hydrogen bonds with the pyridyl N atom and also with a symmetry-related water mol­ecule.

## Related literature

For 3-(4-chloro­phenyl)-1-(3-pyridyl)prop-2-en-1-one, which belongs to a non-centrosymmetric space group, see: Uchida *et al.* (1998 ▶). For the general synthesis by the Claisen–Schmidt condensation, see: Vogel (1999 ▶). For literature on related compounds exhibiting second-harmonic generation activity, see: Gu *et al.* (2008 ▶); Ravindra *et al.* (2008*a* ▶,*b* ▶).
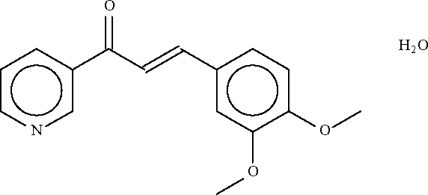


## Experimental

### 

#### Crystal data

C_16_H_15_NO_3_·H_2_O
*M* *_r_* = 287.31
Monoclinic, 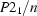

*a* = 17.9809 (4) Å
*b* = 4.5004 (1) Å
*c* = 18.2230 (4) Å
β = 101.775 (2)°
*V* = 1443.60 (6) Å^3^
*Z* = 4
Mo *K*α radiation
μ = 0.10 mm^−1^
*T* = 100 K
0.20 × 0.15 × 0.10 mm

#### Data collection

Bruker SMART APEX diffractometer
Absorption correction: none
12630 measured reflections
3315 independent reflections
2362 reflections with *I* > 2σ*I*)
*R*_int_ = 0.041

#### Refinement

*R*[*F* ^2^ > 2σ(*F* ^2^)] = 0.044
*wR*(*F* ^2^) = 0.126
*S* = 1.02
3315 reflections
200 parameters
2 restraints
H atoms treated by a mixture of independent and constrained refinement
Δρ_max_ = 0.52 e Å^−3^
Δρ_min_ = −0.20 e Å^−3^

### 

Data collection: *APEX2* (Bruker, 2007 ▶); cell refinement: *SAINT* (Bruker, 2007 ▶); data reduction: *SAINT*; program(s) used to solve structure: *SHELXS97* (Sheldrick, 2008 ▶); program(s) used to refine structure: *SHELXL97* (Sheldrick, 2008 ▶); molecular graphics: *X-SEED* (Barbour, 2001 ▶); software used to prepare material for publication: *publCIF* (Westrip, 2009 ▶).

## Supplementary Material

Crystal structure: contains datablocks global, I. DOI: 10.1107/S1600536809013944/bt2930sup1.cif

Structure factors: contains datablocks I. DOI: 10.1107/S1600536809013944/bt2930Isup2.hkl

Additional supplementary materials:  crystallographic information; 3D view; checkCIF report

## Figures and Tables

**Table 1:** Hydrogen-bond geometry (Å, °)

*D*—H⋯*A*	*D*—H	H⋯*A*	*D*⋯*A*	*D*—H⋯*A*
O1*W*—H11⋯N1	0.83 (1)	2.05 (1)	2.857 (2)	163 (2)
O1*W*—H12⋯O1*W*^i^	0.85 (1)	1.94 (3)	2.763 (2)	162 (7)

Symmetry code: (i) 
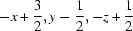
.

**Fig. 1.**
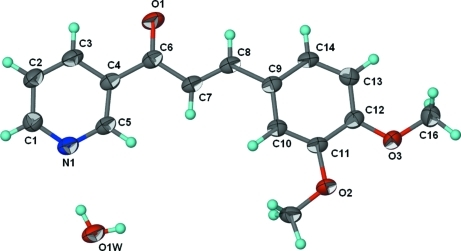
Anisotropic displacement ellipsoid plot (Barbour, 2001) of the title compound at the 70% probability level. H atoms are drawn as spheres of arbitrary radius.

## supplementary crystallographic information

### Comment

Some chalcone derivatives exhibit high second-harmonic generation conversion efficiency (Gu *et al.*, 2008; Ravindra *et al.*, 2008*a*,*b*). The title compound was synthesized for the purpose of examining this property; unfortunately, the compound crystallizes in a centrosymmetric space group.

### Experimental

The compound was synthesized by the Claisen–Schmidt condensation (Vogel, 1999). To a mixture of ethanol (20 ml) and 10% sodium hydroxide solution (5 ml) was added an ethanol (15 ml) solution of 3-acetyl pyridine (0.001 mol) and 3,4-dimethoxybenzaldehyde (0.001 mol). The temperature of the mixture was maintained at below 298 K for 2 h. The solid product that formed was washed with water. The compound was recrystallized from methanol.

### Refinement

Carbon-bound H atoms were placed in calculated positions (C—H 0.95–0.98 Å) and were included in the refinement in the riding model approximation, with *U*(H) constrained to 1.2–1.5*U*_eq_(C). The water H atoms were located in a difference Fourier map, and were refined with a distance restraint of O—H = 0.84 (1) Å; their isotropic temperature factors were refined.

### Crystal data

**Table:**

C_16_H_15_NO_3_·H_2_O	*F*(000) = 608
*M**_r_* = 287.31	*D*_x_ = 1.322 Mg m^−^^3^
Monoclinic, *P*2_1_/*n*	Mo *K*α radiation, λ = 0.71073 Å
Hall symbol: -P 2yn	Cell parameters from 2693 reflections
*a* = 17.9809 (4) Å	θ = 2.3–28.2°
*b* = 4.5004 (1) Å	µ = 0.10 mm^−^^1^
*c* = 18.2230 (4) Å	*T* = 100 K
β = 101.775 (2)°	Prism, yellow
*V* = 1443.60 (6) Å^3^	0.20 × 0.15 × 0.10 mm
*Z* = 4	

### Data collection

**Table:**

Bruker SMART APEX diffractometer	2362 reflections with *I* > 2σ(*I*)
Radiation source: fine-focus sealed tube	*R*_int_ = 0.041
graphite	θ_max_ = 27.5°, θ_min_ = 1.5°
ω scans	*h* = −23→23
12630 measured reflections	*k* = −5→5
3315 independent reflections	*l* = −23→23

### Refinement

**Table:**

Refinement on *F*^2^	Primary atom site location: structure-invariant direct methods
Least-squares matrix: full	Secondary atom site location: difference Fourier map
*R*[*F*^2^ > 2σ(*F*^2^)] = 0.044	Hydrogen site location: inferred from neighbouring sites
*wR*(*F*^2^) = 0.126	H atoms treated by a mixture of independent and constrained refinement
*S* = 1.02	*w* = 1/[σ^2^(*F*_o_^2^) + (0.0628*P*)^2^ + 0.3539*P*] where *P* = (*F*_o_^2^ + 2*F*_c_^2^)/3
3315 reflections	(Δ/σ)_max_ = 0.001
200 parameters	Δρ_max_ = 0.52 e Å^−^^3^
2 restraints	Δρ_min_ = −0.20 e Å^−^^3^

### Fractional atomic coordinates and isotropic or equivalent isotropic displacement parameters (Å^2^)

**Table:**

	*x*	*y*	*z*	*U*_iso_*/*U*_eq_	
O1	0.41877 (6)	0.8369 (3)	0.27622 (7)	0.0397 (3)	
O2	0.80564 (6)	0.3782 (3)	0.50243 (6)	0.0340 (3)	
O3	0.76194 (6)	0.0474 (3)	0.59966 (6)	0.0332 (3)	
O1W	0.74070 (7)	1.5349 (3)	0.20508 (8)	0.0402 (3)	
H11	0.6951 (6)	1.524 (5)	0.1836 (11)	0.059 (7)*	
H12	0.748 (4)	1.409 (13)	0.241 (3)	0.27 (3)*	
N1	0.58178 (7)	1.4202 (3)	0.15840 (8)	0.0308 (3)	
C1	0.52118 (9)	1.5143 (4)	0.10854 (9)	0.0316 (4)	
H1	0.5290	1.6520	0.0713	0.038*	
C2	0.44804 (9)	1.4221 (4)	0.10827 (10)	0.0351 (4)	
H2	0.4066	1.4938	0.0716	0.042*	
C3	0.43629 (8)	1.2243 (4)	0.16218 (9)	0.0313 (4)	
H3	0.3864	1.1582	0.1633	0.038*	
C4	0.49781 (8)	1.1211 (3)	0.21511 (8)	0.0251 (3)	
C5	0.56925 (8)	1.2269 (4)	0.21027 (9)	0.0279 (4)	
H5	0.6118	1.1577	0.2460	0.034*	
C6	0.48429 (8)	0.9066 (4)	0.27391 (9)	0.0271 (4)	
C7	0.54975 (8)	0.7857 (4)	0.32741 (9)	0.0274 (4)	
H7	0.5996	0.8515	0.3257	0.033*	
C8	0.53984 (8)	0.5844 (4)	0.37843 (9)	0.0265 (3)	
H8	0.4889	0.5264	0.3777	0.032*	
C9	0.59780 (8)	0.4441 (3)	0.43488 (8)	0.0244 (3)	
C10	0.67609 (8)	0.4868 (4)	0.43855 (9)	0.0260 (3)	
H10	0.6927	0.6099	0.4027	0.031*	
C11	0.72834 (8)	0.3517 (4)	0.49363 (9)	0.0262 (3)	
C12	0.70494 (8)	0.1676 (3)	0.54753 (9)	0.0267 (4)	
C13	0.62805 (9)	0.1256 (4)	0.54443 (9)	0.0279 (4)	
H13	0.6114	0.0045	0.5806	0.034*	
C14	0.57558 (8)	0.2614 (4)	0.48808 (9)	0.0267 (3)	
H14	0.5230	0.2288	0.4858	0.032*	
C15	0.83247 (9)	0.5723 (4)	0.45185 (10)	0.0317 (4)	
H15A	0.8881	0.5795	0.4644	0.048*	
H15B	0.8120	0.7721	0.4558	0.048*	
H15C	0.8158	0.4988	0.4005	0.048*	
C16	0.74125 (10)	−0.1299 (4)	0.65737 (10)	0.0365 (4)	
H16A	0.7872	−0.1916	0.6929	0.055*	
H16B	0.7136	−0.3063	0.6350	0.055*	
H16C	0.7088	−0.0136	0.6838	0.055*	

### Atomic displacement parameters (Å^2^)

**Table:**

	*U*^11^	*U*^22^	*U*^33^	*U*^12^	*U*^13^	*U*^23^
O1	0.0184 (6)	0.0609 (9)	0.0394 (7)	−0.0013 (5)	0.0049 (5)	0.0074 (6)
O2	0.0173 (5)	0.0427 (7)	0.0417 (7)	0.0009 (5)	0.0052 (5)	0.0096 (6)
O3	0.0228 (5)	0.0391 (7)	0.0363 (7)	0.0030 (5)	0.0029 (5)	0.0096 (5)
O1W	0.0225 (6)	0.0420 (7)	0.0526 (8)	−0.0010 (5)	−0.0007 (6)	0.0041 (7)
N1	0.0228 (6)	0.0373 (8)	0.0318 (7)	−0.0007 (6)	0.0042 (5)	−0.0019 (6)
C1	0.0300 (8)	0.0321 (9)	0.0312 (9)	−0.0011 (7)	0.0031 (7)	0.0003 (7)
C2	0.0250 (8)	0.0379 (10)	0.0383 (10)	0.0003 (7)	−0.0034 (7)	0.0035 (8)
C3	0.0200 (7)	0.0346 (9)	0.0368 (9)	−0.0010 (7)	0.0005 (7)	−0.0015 (8)
C4	0.0205 (7)	0.0283 (8)	0.0256 (8)	0.0018 (6)	0.0030 (6)	−0.0062 (6)
C5	0.0202 (7)	0.0346 (9)	0.0280 (8)	0.0030 (6)	0.0027 (6)	−0.0035 (7)
C6	0.0215 (7)	0.0328 (9)	0.0267 (8)	0.0023 (6)	0.0038 (6)	−0.0050 (7)
C7	0.0198 (7)	0.0317 (9)	0.0295 (8)	0.0003 (6)	0.0027 (6)	−0.0043 (7)
C8	0.0195 (7)	0.0307 (8)	0.0289 (8)	0.0004 (6)	0.0036 (6)	−0.0069 (7)
C9	0.0215 (7)	0.0244 (8)	0.0268 (8)	−0.0004 (6)	0.0036 (6)	−0.0063 (6)
C10	0.0218 (7)	0.0276 (8)	0.0290 (8)	−0.0001 (6)	0.0063 (6)	−0.0018 (7)
C11	0.0181 (7)	0.0289 (8)	0.0320 (8)	−0.0002 (6)	0.0060 (6)	−0.0028 (7)
C12	0.0230 (7)	0.0264 (8)	0.0298 (8)	0.0026 (6)	0.0030 (6)	−0.0027 (7)
C13	0.0264 (8)	0.0271 (8)	0.0308 (8)	−0.0021 (6)	0.0069 (6)	−0.0013 (7)
C14	0.0194 (7)	0.0279 (8)	0.0328 (8)	−0.0021 (6)	0.0056 (6)	−0.0038 (7)
C15	0.0213 (7)	0.0362 (9)	0.0386 (9)	−0.0026 (7)	0.0085 (7)	0.0014 (8)
C16	0.0334 (9)	0.0400 (10)	0.0357 (9)	0.0040 (8)	0.0059 (7)	0.0078 (8)

### Geometric parameters (Å, °)

**Table:**

O1—C6	1.2282 (18)	C7—C8	1.336 (2)
O2—C11	1.3714 (17)	C7—H7	0.9500
O2—C15	1.4237 (19)	C8—C9	1.451 (2)
O3—C12	1.3596 (18)	C8—H8	0.9500
O3—C16	1.429 (2)	C9—C14	1.390 (2)
O1W—H11	0.84 (1)	C9—C10	1.409 (2)
O1W—H12	0.85 (1)	C10—C11	1.369 (2)
N1—C1	1.337 (2)	C10—H10	0.9500
N1—C5	1.337 (2)	C11—C12	1.413 (2)
C1—C2	1.378 (2)	C12—C13	1.385 (2)
C1—H1	0.9500	C13—C14	1.387 (2)
C2—C3	1.374 (2)	C13—H13	0.9500
C2—H2	0.9500	C14—H14	0.9500
C3—C4	1.391 (2)	C15—H15A	0.9800
C3—H3	0.9500	C15—H15B	0.9800
C4—C5	1.390 (2)	C15—H15C	0.9800
C4—C6	1.499 (2)	C16—H16A	0.9800
C5—H5	0.9500	C16—H16B	0.9800
C6—C7	1.471 (2)	C16—H16C	0.9800
			
C11—O2—C15	116.54 (12)	C14—C9—C8	118.98 (13)
C12—O3—C16	117.66 (12)	C10—C9—C8	122.71 (14)
H11—O1W—H12	108 (5)	C11—C10—C9	120.24 (15)
C1—N1—C5	117.06 (13)	C11—C10—H10	119.9
N1—C1—C2	123.50 (16)	C9—C10—H10	119.9
N1—C1—H1	118.3	C10—C11—O2	125.07 (14)
C2—C1—H1	118.3	C10—C11—C12	120.85 (14)
C3—C2—C1	118.58 (15)	O2—C11—C12	114.08 (13)
C3—C2—H2	120.7	O3—C12—C13	125.32 (14)
C1—C2—H2	120.7	O3—C12—C11	115.47 (13)
C2—C3—C4	119.73 (15)	C13—C12—C11	119.21 (14)
C2—C3—H3	120.1	C12—C13—C14	119.52 (15)
C4—C3—H3	120.1	C12—C13—H13	120.2
C5—C4—C3	117.15 (15)	C14—C13—H13	120.2
C5—C4—C6	123.58 (14)	C13—C14—C9	121.87 (14)
C3—C4—C6	119.27 (14)	C13—C14—H14	119.1
N1—C5—C4	123.99 (14)	C9—C14—H14	119.1
N1—C5—H5	118.0	O2—C15—H15A	109.5
C4—C5—H5	118.0	O2—C15—H15B	109.5
O1—C6—C7	121.76 (15)	H15A—C15—H15B	109.5
O1—C6—C4	119.06 (14)	O2—C15—H15C	109.5
C7—C6—C4	119.17 (13)	H15A—C15—H15C	109.5
C8—C7—C6	120.64 (14)	H15B—C15—H15C	109.5
C8—C7—H7	119.7	O3—C16—H16A	109.5
C6—C7—H7	119.7	O3—C16—H16B	109.5
C7—C8—C9	127.59 (14)	H16A—C16—H16B	109.5
C7—C8—H8	116.2	O3—C16—H16C	109.5
C9—C8—H8	116.2	H16A—C16—H16C	109.5
C14—C9—C10	118.31 (14)	H16B—C16—H16C	109.5
			
C5—N1—C1—C2	−0.1 (2)	C14—C9—C10—C11	−0.3 (2)
N1—C1—C2—C3	0.4 (3)	C8—C9—C10—C11	179.17 (14)
C1—C2—C3—C4	−0.3 (3)	C9—C10—C11—O2	−179.94 (14)
C2—C3—C4—C5	0.1 (2)	C9—C10—C11—C12	0.1 (2)
C2—C3—C4—C6	179.67 (15)	C15—O2—C11—C10	2.9 (2)
C1—N1—C5—C4	−0.2 (2)	C15—O2—C11—C12	−177.12 (14)
C3—C4—C5—N1	0.2 (2)	C16—O3—C12—C13	−1.9 (2)
C6—C4—C5—N1	−179.40 (15)	C16—O3—C12—C11	177.32 (14)
C5—C4—C6—O1	176.30 (15)	C10—C11—C12—O3	−179.61 (14)
C3—C4—C6—O1	−3.3 (2)	O2—C11—C12—O3	0.4 (2)
C5—C4—C6—C7	−3.6 (2)	C10—C11—C12—C13	−0.3 (2)
C3—C4—C6—C7	176.84 (14)	O2—C11—C12—C13	179.69 (14)
O1—C6—C7—C8	3.0 (2)	O3—C12—C13—C14	−179.96 (14)
C4—C6—C7—C8	−177.10 (14)	C11—C12—C13—C14	0.8 (2)
C6—C7—C8—C9	−179.94 (14)	C12—C13—C14—C9	−1.1 (2)
C7—C8—C9—C14	173.53 (15)	C10—C9—C14—C13	0.8 (2)
C7—C8—C9—C10	−5.9 (3)	C8—C9—C14—C13	−178.66 (14)

### Hydrogen-bond geometry (Å, °)

**Table:**

*D*—H···*A*	*D*—H	H···*A*	*D*···*A*	*D*—H···*A*
O1W—H11···N1	0.83 (1)	2.05 (1)	2.857 (2)	163 (2)
O1W—H12···O1W^i^	0.85 (1)	1.94 (3)	2.763 (2)	162 (7)

Symmetry codes: (i) −*x*+3/2, *y*−1/2, −*z*+1/2.
